# Deficiency of the Metalloproteinase-Disintegrin ADAM8 Is Associated with Thymic Hyper-Cellularity

**DOI:** 10.1371/journal.pone.0012766

**Published:** 2010-09-15

**Authors:** Klaus Gossens, Silvia Naus, Georg A. Holländer, Hermann J. Ziltener

**Affiliations:** 1 The Biomedical Research Centre, University of British Columbia, Vancouver, British Columbia, Canada; 2 Department of Pathology and Laboratory Medicine, University of British Columbia, Vancouver, British Columbia, Canada; 3 Laboratory of Pediatric Immunology, Department of Biomedicine, University of Basel and The University Children's Hospital (UKBB), Basel, Switzerland; New York University, United States of America

## Abstract

**Background:**

Thymopoiesis requires thymocyte-stroma interactions and proteases that promote cell migration by degrading extracellular matrix and releasing essential cytokines and chemokines. A role for several members of the A Disintegrin and Metalloprotease (ADAM) family in T cell development has been reported in the past.

**Methodology/Principal Findings:**

Here, we present data indicating that the family member ADAM8 plays a role in thymic T cell development. We used qrtPCR on FACS sorted thymic subsets together with immunofluorescene to analyze thymic ADAM8 expression. We found that ADAM8 was expressed in murine thymic stromal cells and at lower levels in thymocytes where its expression increased as cell matured, suggesting involvement of ADAM8 in thymopoiesis. Further flow cytometry analysis revealed that ADAM8 deficient mice showed normal development and expansion of immature thymocyte subsets. There was however an intrathymic accumulation of single positive CD4 and CD8 T cells which was most noticeable in the late mature T cell subsets. Accumulation of single positive T cells coincided with changes in the thymic architecture manifest in a decreased cortex/medulla ratio and an increase in medullary epithelial cells as determined by histology and flow cytometry. The increase in single positive T cells was thymus-intrinsic, independent of progenitor homing to the thymus or thymic exit rate of mature T cells. Chemotaxis assays revealed that ADAM8 deficiency was associated with reduced migration of single positive thymocytes towards CCL21.

**Conclusions/Significance:**

Our results show that ADAM8 is involved in T cell maturation in the medulla and suggest a role for this protease in fine-tuning maturation of thymocytes in the medulla. In contrast to ADAM10 and ADAM17 lack of ADAM8 appears to have a relatively minor impact on T cell development, which was unexpected given that maturation of thymocytes is dependent on proper localization and timing of migration.

## Introduction

Thymic development of T cells is characterized by a series of complex processes including gene rearrangement, positive and negative selection for T cell receptor specificity, proliferation and differentiation (Reviewed in [1]). T cell precursors enter the thymus from the blood near the cortico-medullary junction, migrate into the cortex and subsequently undergo four successive CD4/CD8 double negative (DN) stages: DN1 (CD44^+^, CD25^−^), DN2 (CD44^+^, CD25^+^), DN3 (CD44^−^, CD25^+^) and pre-double positive (pre-DP; (CD44^−^, CD25^−^). At the DN2/DN3 stages, the β-chain of the TCR is re-arranged. After successful β-chain selection, DN thymocytes mature into CD4/CD8 double positive (DP) thymocytes that accumulate in the cortex. Upon rearrangement of the α-chain of the TCR thymocytes undergo positive selection that is followed by a down-regulation of one of the TCR co-receptors CD4 or CD8 according to the MHC restriction of the TCR. Single positive (SP) CD4 or CD8 thymocytes migrate to the medulla where they undergo negative selection, a process controlled by medullary thymic epithelial and dendritic cells that removes cells expressing auto-reactive TCR. Mature self-tolerant thymocytes leave the thymus for the periphery near the cortico-medullary junction.

Development and maturation of T cells is associated with intrathymic migration such that specific developmental stages can be allocated to particular thymic areas [2], [3]. These interactions are highly dependent on thymocyte-stroma contacts and involve enzymatic activities of metalloproteinases that enable cells to migrate on a cellular substrate by remodeling the extracellular matrix (ECM) and/or by releasing cytokines, chemokines or growth factors [4], [5]. A general function for metalloproteinases in thymic T cell development was determined by assessing the effect of a broad-spectrum metalloproteinase inhibitor. In fetal thymic organ cultures, the inhibitor hindered thymocyte development at the early DN stages, decreased TCR-β gene rearrangement and reduced the numbers of DP and SP thymocytes [6]. A role for several members of A Disintegrin And Metalloproteinase (ADAM) family in thymic T cell development has been reported [7], [8], [9], [10]. The ADAMs form a widely expressed family of membrane-anchored metalloproteinases with multiple domains including a zinc-dependent metalloproteinase domain and a disintegrin/cysteine-rich domain and with functions in physiological processes such as sperm-egg fusion, neovascularization, and neurogenesis [11]. Consistent with the above-mentioned findings by Haidl et al. [6], the expression of a dominant negative form of ADAM10 in transgenic mice as well as the expression of a catalytically inactive form of ADAM17 resulted in a partial block at the transition from the DN to DP stage causing a lower thymic cellularity. Both ADAM10 and ADAM17 are believed to influence T cell development via their activity as sheddases for Notch receptor and Notch ligands [8], [9], [10]. The prominent expression in murine purified fetal thymic epithelial cells (TEC) as well as in adult TEC indicated also a role for ADAM28 in thymic T cell development [7]. However, it remains elusive whether ADAM28 contributes to TEC-TEC or TEC-thymocyte interaction via the disintegrin/cysteine-rich domain or whether it is involved in release of cell surface proteins like ADAM10 and ADAM17 [7]. The family member ADAM8/CD156/M2S has previously been detected in fetal and adult thymi [12], [13]. Despite its prominent expression in most immune cells and tissues particularly under inflammatory conditions [14], [15], [16], [17], [18], the role of ADAM8 in thymus biology has not yet been examined. Here, we show that ADAM8 is broadly expressed by thymic stromal cells and is detected in mature thymocytes only at lower levels. Thymi deficient for ADAM8 expression are hypertrophic due to an increase of SP thymocytes that results in an altered thymic architecture. A reduced migration towards CCL21, increased cell proliferation and a decreased rate of apoptosis are likely causes for the accumulation of thymocytes in ADAM8 deficient thymi.

## Materials and Methods

### Ethics Statement

All animal experiments were performed according to institutional guidelines and approved by the Animal Care Committee of the University of British Columbia.

### Mice

C57Bl/6 (CD45.2), congenic C57Bl/6 (CD45.1) and IL-7Rα-chain receptor deficient mice were purchased from Jackson Laboratory. ADAM8 deficient mice backcrossed on C57Bl/6 background [13] were obtained from Jörg W. Bartsch (King's College London, UK). All mice were bred and maintained in the specific pathogen free (SPF) animal facility in the Biomedical Research Centre.

### Tissue preparation

Single cell suspensions of peripheral lymph nodes and spleens were prepared by squeezing tissues through a mesh. For bone marrow suspensions, femurs and tibias were flushed with RPMI, 2% FCS, 2 mM EDTA and cells separated by pipetting. Thymi were squeezed between two frosted glass slides and washed with RPMI, 2% FCS, 2 mM EDTA. The thymocyte containing suspension fractions were filtered and kept for flow cytometry analysis. For the preparation of thymic stromal cells, thymi were minced and incubated with Dispase/Collagenase (Roche) as previously described [19].

### Quantitative Real-Time PCR (qrtPCR)

RNA was extracted using TRIzol solution (Invitrogen) and 1 µg of DNAse-treated total RNA was reverse-transcribed using Superscript III (Invitrogen). Gene expression for ADAM8 was quantified using quantitative real time PCR for ADAM8 (forward 5′-GTGTGGTTGTGGTCTTGGTG-3′, reverse 5′-CTTGGGTGCCACACTCCT-3′) and the endogenous control HPRT (forward 5′-GCTGGTGAAAAGGACCTCT-3′, reverse 5′-CACAGGACTAGAACACCTGC-3′). Reactions were performed using SYBR green master mix (Bioline). All reactions were run on a Rotor Gene 6000 real-time PCR system (Corbet Life Science). Relative mRNA expression was calculated using the REST software tool [20].

### Flow cytometry and cell sorting

Cells were stained in buffer (PBS, 2% FCS, 2 mM EDTA) with titrated amounts of antibodies: CD3 (145-2C11, Hybridoma Bank), CD4 (RM4-5, eBioscience), CD8 (53-6.7, eBioscience), CD11b (M1/70, Hybridoma Bank), CD11c (N418, Hybridoma Bank), CD19 (ID3, Hybridoma Bank), panNK (DX5, Hybridoma Bank), γδ-TCR (eBioGl3, eBioscience), Gr-1 (RB6-8C5, Hybridoma Bank), Ter119 (Ter119, Hybridoma Bank), CD45.1 (A20, eBioscience), CD45.2 (104, eBioscience), CD24 (M1/69, eBioscience), L-Selectin (Mel14, Hybridoma Bank), CD25 (PC61, eBioscience), CD44 (IM7, Hybridoma Bank), CD45 (30-F11, eBioscience) Sca-1 (D7, BD Biosciences), CD31 (390, BD Biosciences), CD144 (eBioBV13, eBioscience), MHCII (AF6-120.1, BioLegend), Ly51 (6C3, BD), EpCAM (G8.8, eBioscience), UEA-1 (Vector Laboratory), cleaved Caspase-3 (Cell Signaling), Ki-67 (Santa Cruz Biotechnology). For the detection of lineage negative cells (Lin^−^), suspension cells were stained with an antibody mix of lineage markers: CD3, CD4, CD8, CD11b, CD11c, CD19, panNK, γδ-TCR, Gr-1, Ter119. For TCR staining, the Mouse Vβ TCR Screening Panel was used according to manufacturer's instructions and furthermore a set of antibodies directed against Vα 2 TCR (B20.1), Vα 3.2[b, c] TCR (RR3-16), Vα 8.3 TCR (R21.14) and Vα 11.1, 11.2[b, d] TCR (RR8-1) (all BD Biosciences). For intracellular Caspase-3 staining, thymocytes were cell-surface stained and fixed with 0.5% PFA (10 minutes, 37°C) and subsequently treated with 90% methanol (30 minutes, 4°C) prior to intracellular staining. For Ki-67 detection, thymocytes were cell-surface stained and then fixed with 70% ice-cold ethanol over night prior to intracellular staining. CCR7 was detected with CCL19-Fc (eBioscience) according to the manufacturer's recommendation.

Where required, a fixed number of reference beads (Interfacial Dynamics, Invitrogen) were added to each sample to quantify cellularity. Samples were analyzed on a Calibur or LSRII flow cytometer and cell sorting was performed on a FACS Vantage or FACS Aria (all BD Bioscience). Data were analyzed with FlowJo software (Treestar).

### Immunofluorescence

Frozen thymic sections (8 µm) were stored at −80°C until used. After rehydration in washing buffer (PBS, 0.1% TritonX, 0.05% Tween20), sections were fixed in acetone (6 minutes) and blocked with 20% goat serum in washing buffer to reduce unspecific binding. Sections were incubated with titrated amounts of primary anti β-galactosidase antibody (ab9361, Abcam), followed by incubation with antibodies directed against β5t (MBL), MTS10 (kindly provided by Dr. Richard Boyd), ER-TR7 (kindly provided by Dr. Willem van Ewijk) and CD3 (KT3, Hybridoma Bank), respectively. Alexa Fluor conjugates (Invitrogen) were used as secondary reagents. Sections were mounted using Hydromount (National Diagnostics). Images were acquired using a Zeiss LSM510 Meta confocal microscope and analyzed using ImageJ1.43k (NIH).

### Assessment of cortex/medulla (C/M) ratios

Thymic sections (20 µm) were fixed (4% PFA/PBS, 15 minutes), stained for 15 minutes in Harris' Alum Hematoxylin (Harleco) and subsequently washed for 5 minutes in tap water. Sections were then stained for 5 minutes with 0.25% Eosin Y (EMD), 0.5% glacial acetic acid in Ethanol. After consecutive dehydration in 80%, 90%, 100% ethanol and xylene, sections were mounted with Permount (Fisher Chemicals). Slides were analyzed on a dissecting microscope (Leica) and pictures acquired using QCapture software (QIaging). Image segmentation to determine cortex/medulla ratio was done by thresholding using ImageJ1.43k (NIH) software.

### Short-term receptivity

Short-term receptivity was determined by i.v. injection of fluorescent-labeled cells, as previously described [21]. In brief, 40×10^6^ unfractionated CFSE-labeled bone marrow cells were i.v. injected into recipients and the numbers of CFSE-labeled cells in thymi were determined 24 hours later by flow cytometry.

### Migration assay

Transwell chambers (5 µm, Costar) were preincubated for 1 hour with media (RPMI supplemented with 0.5% BSA) at 37°C. Thymocytes (2×10^6^) in 100 µL media were placed in the upper wells of the transwell chambers and 600 µl media with or without chemokine (CCL21, 100 nM) added to the lower chambers. After 90 minutes at 37°C, cells in the lower and upper chambers were harvested, stained with anti-CD4 and CD8 antibodies and analyzed by flow cytometry. Where indicated, migration inserts had been precoated on both sides with Fibronectin (20 µg/mL in PBS, 2 hours, room temperature, Calbiochem) and had then been dried before preincubation.

### Intrathymic FITC labeling

Intrathymic FITC labeling was performed as previously reported [22]. 10 µl FITC solution (2 mg/mL in PBS, Sigma Aldrich) were injected into each thymic lobe of anesthetized mice using a 1 mL syringe with an attached 28G needle mounted on a Stepper (Tridak). Thymic labeling efficiency was on average 75%. Mice were sacrificed 36 hours after injection and the numbers of labeled CD4 and CD8 cells quantified both in the thymus and lymph nodes using flow cytometry.

### Intrathymic bone marrow injection

Intrathymic bone marrow injection was performed as previously described [23]. Bone marrow cells (2×10^6^) in 10 µL RPMI were injected into each thymic lobe of anesthetized mice using a 1 mL syringe with an attached 28G needle mounted on a Stepper (Tridak). Ten days later, chimerism of bone marrow cells was assessed by flow cytometry.

### Statistics

Data are presented as mean values and error bars as SEM. Statistical significance was assessed using unpaired, two-tailed, student's t test with * p<0.05, ** p<0.01 and *** p<0.001. Statistics were calculated using SPSS 16.0 for Mac (SPSS Inc.).

## Results

### ADAM8 is expressed in thymic stromal cells and thymocytes

It has previously been shown that members of the ADAM family of metalloproteinases are expressed in adult thymi [7], [8], [9], [10], [12], [13] and that ADAM8 is expressed in fetal and adult thymi [12], [13]. We therefore intended to characterize the function of ADAM8 in the adult murine thymus comparing wild type (wt) and ADAM8 deficient (ADAM8^−/−^) mice.

**Figure 1:**
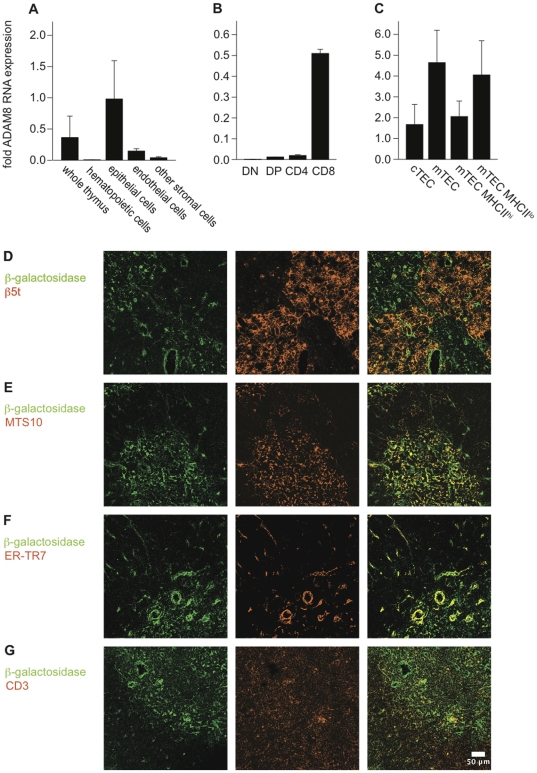
ADAM8 is expressed in thymic stromal cells and thymocytes. Relative ADAM8 RNA levels as determined by qrtPCR in (A) total thymic tissue, thymic hematopoietic cells (CD45^+^, EpCAM^−^), epithelial cells (CD45^−^, EpCAM^+^), endothelial cells (CD45^−^, EpCAM^−^, CD31^+^) and other non-epithelial stromal cells (EpCAM^−^, CD45^−^, CD31^−^), (B) thymic T cell subsets and (C) TEC. FACS sorted cells (>95% purity) were analyzed for ADAM8 RNA expression using the reference gene HPRT. ADAM8 RNA values are expressed relative to HPRT expression. Data are presented as mean values and error bars as SEM. Data are from at least two independent experiments (n≥3). (D–G) Reporter gene (β-galactosidase) expression in ADAM8^−/−^ thymic sections detected by immunofluorescence. Thymic sections were stained with antibodies directed against β-galactosidase to reveal potential ADAM8 expression and (D) β5t marking cTEC, (E) MTS10 marking mTEC, (F) ER-TR7 marking fibroblasts and (G) KT-3 marking T cells. Scales bar 50 µm. Data are from at least four independent experiments (n≥4).

We used qrtPCR on highly enriched thymic cells (purity >95%) to detect ADAM8 mRNA expression (Figure 1A–C). ADAM8 mRNA was predominantly expressed in epithelial cells (CD45^−^ EpCAM^+^) whereas endothelial cells (CD45^−^, EpCAM^−^, CD31^+^) non-epithelial stromal cells (EpCAM^−^, CD45^−^, CD31^−^) and hematopoietic cells (CD45^+^ EpCAM^−^) expressed only 15%, 4% and 1% thereof, respectively (Figure 1A). ADAM8 RNA expression levels in thymocytes were lowest in DN cells and highest in CD8 cells (Figure 1B). In the epithelial cell compartment, ADAM8 expression was about three times higher in medullary TEC (mTEC: CD45^−^, EpCAM^+^, Ly51^lo^, UEA-1^hi^) as compared to cortical TEC (cTEC; CD45^−^, EpCAM^+^, Ly51^hi^, UEA-1^lo^) (Figure 1C) confirming published results [12]. mTEC with a mature phenotype expressing high levels of MHCII expressed 50% of the ADAM8 mRNA levels of immature mTEC expressing low levels of MHCII.

The ADAM8^−/−^ mice used in our studies contain a β-galactosidase coding sequence with an internal ribosomal entry site in the ADAM8 locus, providing an opportunity to analyze ADAM8 expression in thymic sections. Antibody staining of β-galactosidase revealed a strong expression in mTEC (Fig. 1E) as defined by MTS10^+^ labeling and weak expression in cTEC (Fig. 1D) as defined by β5t^+^ labeling consistent with our qrtPCR data. Since the qrtPCR data suggested ADAM8 expression in non-epithelial cells we stained for ER-TR7 to label an antigen produced by reticular fibroblasts. Indeed, β-galactosidase was also detected in ER-TR7^+^ cells of the thymic conduit network. Similarly, β-galactosidase was expressed in CD3^+^ cells confirming our RNA expression profiling (Fig. 1G).

Taken together, qrtPCR and histological analyses identified ADAM8 to be predominantly expressed in the medulla by mTEC and, though at lesser degree, by CD8 thymocytes that typically reside in this compartment.

### ADAM8^−/−^ thymi are increased in weight and contain increased numbers of mature thymocytes

**Figure 2:**
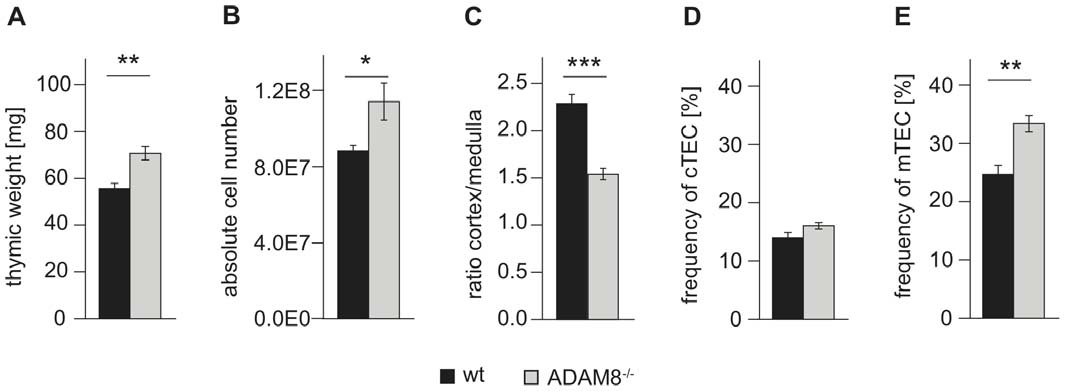
ADAM8^−/−^ thymi have an increased mass and a reduced cortex/medulla ratio associated with a relative increase in mTEC. (A) Weight and (B) total numbers of thymocytes thymi from age- and gender-matched wt and ADAM^−/−^ mice (n = 8). (C) Cortex/medulla ratios examined on Hematoxylin/Eosin stained 20 µm sections of wt and ADAM8^−/−^ thymi (each 6 sections of 6 wt or ADAM^−/−^ thymi). Frequencies of (D) cTEC (Ly51^hi^, UEA-1^lo^) and (E) mTEC (Ly51^lo^, UEA-1^hi^) in CD45^−^, EpCAM^+^ thymic epithelial fraction of thymic suspensions obtained by collagenase/dispase treatment. Data are representative of two independent experiments with at least four mice per group. Data are presented as mean values and error bars as SEM. *, p<0.05, **, p<0.01, *** p<0.001.

Adult ADAM8^−/−^ thymi had about a 25% higher thymic weight when compared to age-matched wt controls (Figure 2A). This increase in thymic weight was associated with a significant increase in thymic cellularity (Figure 2B). Furthermore, Hematoxylin/Eosin staining and subsequent assessment of the ratio of thymic cortex/medulla area (C/M) comparing tissue sections of wt and ADAM8^−/−^ mice revealed a reduced C/M ratio for ADAM8^−/−^ thymi (Figure 2C) suggesting an increased medulla content compared to cortical tissue in ADAM8^−/−^ mice. Similarly, the relative numbers of cTEC as determined by flow cytometry were comparable for wt and ADAM8^−/−^ thymi, whereas the corresponding values for mTEC were significantly higher for ADAM8^−/−^ mice (Figure 2D,E).

**Figure 3:**
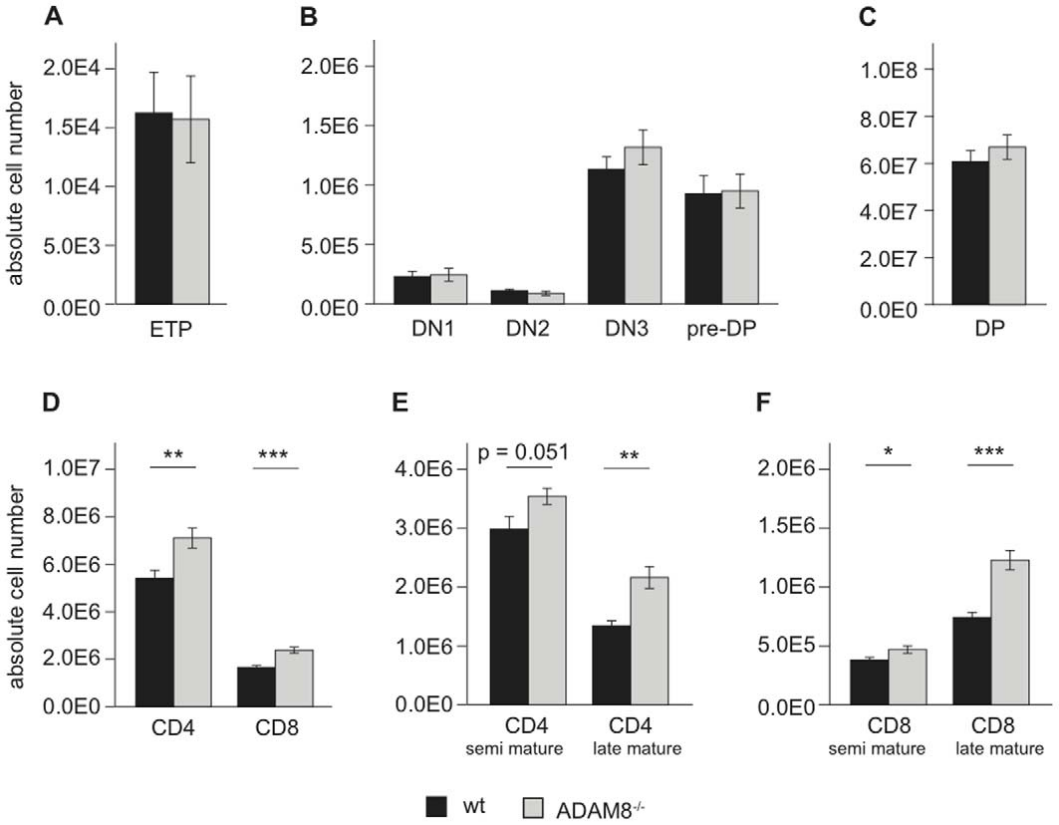
ADAM8^−/−^ thymi have increased numbers of thymocytes. Numbers of (A) early thymic progenitors (ETP: DN1 ckit^+^), (B) DN thymocytes, (C) DP thymocytes, (D) CD4 and CD8 thymocytes, (E) semi and late mature CD4 thymocytes and (F) semi and late mature CD8 thymocytes in wt and ADAM8^−/−^ thymi. Data are representative of 4 independent experiments with n≥4 age- and gender-matched mice per group in each experiment. Data are presented as mean values and error bars as SEM. *, p<0.05, **, p<0.01; *** p<0.001.

Next, we quantified the thymic subpopulations by flow cytometry and found comparable cell numbers of early thymic progenitors (ETP: Lin^−^, CD25^−^, CD44^+^, ckit^+^;), all DN subsets (DN1: Lin^−^, CD44^+^, CD25^−^; DN2: Lin^−^, CD44^+^, CD25^+^; DN3: Lin^−^, CD44^−^, CD25^+^; pre-DP: Lin^−^, CD44^−^, CD25^−^; Figure 3B) and DP thymocytes (Figure 3C). In contrast, the cellularities of CD4 and CD8 thymocytes were significantly increased (p<0.001) in ADAM8^−/−^ compared to wt mice (Figure 3D). This increase was more pronounced for SP thymocytes of late mature (SP CD24^low^ L-Selectin^high^) than of the semi mature stages (SP CD24^high^ L-Selectin^int^; Figure 3E–F). The accumulation of these cells coincided with differential ADAM8 expression and a reduced C/M ratio for ADAM8^−/−^ thymi.

### The accumulation of thymocytes in ADAM8^−/−^ mice is thymus-intrinsic

**Figure 4:**
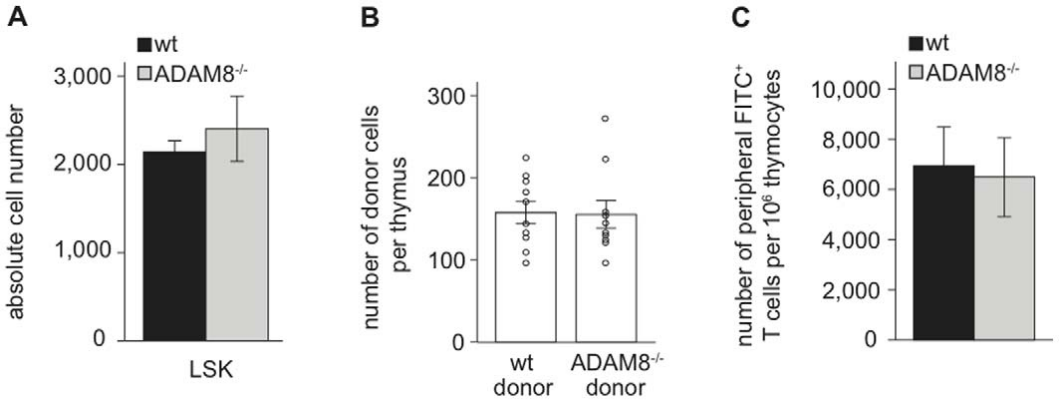
Thymic import and export rates are normal in ADAM8^−/−^ mice. (A) Numbers of LSK cells in wt and ADAM8^−/−^ femurs and tibias. (B) Total numbers of donor cells per thymus after short-term homing of wt and ADAM8^−/−^ bone marrow cells in IL7R^−/−^ mice. (C) Numbers of FITC^+^ CD4 and CD8 lymph node T cells per 10^6^ FITC^+^ CD4 and CD8 thymocytes in wt and ADAM8^−/−^ mice 36 hours after intrathymic injection of FITC. Data in (A) and (B) are representative of two independent experiments and data in (C) of three independent experiments with n≥4 age- and gender-matched mice per group in each experiment. Data are presented as mean values and error bars as SEM.

Generally, an increase in thymocyte numbers and thymic weight can be caused by higher numbers of T cell progenitors entering the thymus and/or reduced thymic exit of mature thymocytes. To determine whether loss of ADAM8 expression affects the size of the hematopoietic stem cell pool, we determined absolute numbers of lineage marker negative, Sca-1^+^, cKit^+^ (LSK) cells known to contain the hematopoietic stem cell fraction [24] in the bone marrow of femurs and tibias of wt and ADAM8^−/−^ mice. The absolute numbers of LSK cells were similar, suggesting that also the numbers of T cell progenitors available to seed the thymus are identical in both strains (Figure 4A). We next determined whether lack of ADAM8 altered thymic homing of cells using a short-term homing assay. For this, CFSE-labeled bone marrow cells from wt or ADAM8^−/−^ donor mice were i.v. injected into IL-7Rα-chain receptor deficient mice (IL7R^−/−^) that are very receptive for exogenous T cell progenitors due to an early block in endogenous T cell development [25], [26], [27]. Twenty-four hours later, recipients were sacrificed and the numbers of donor-derived cells in the thymi were determined by flow cytometry. wt and ADAM8^−/−^ donor cells were detected in similar absolute numbers indicating that an absence of ADAM8 on progenitor cells does not affect their thymus homing capacities (Figure 4B). We next analyzed whether thymic export is impaired in ADAM8^−/−^ mice. wt and ADAM8^−/−^ recipients were intrathymically injected with FITC solution and their lymph nodes analyzed 36 hours later for the presence of FITC-labeled T cells [22]. Using labeling efficiency and thymic cellularity to normalize the data, we found no difference in the export rates of mature thymocytes when comparing wt and ADAM8^−/−^ mice (Figure 4C).

Collectively, these data argue that neither quantity and quality of the T cell precursors nor exit of mature thymocytes is affected in ADAM8^−/−^ mice, and hence suggest that thymus intrinsic processes may be the underlying cause for the increased thymic cellularity.

### ADAM8^−/−^ thymocytes have a higher proliferation and a lower apoptosis rate than wt thymocytes

**Figure 5:**
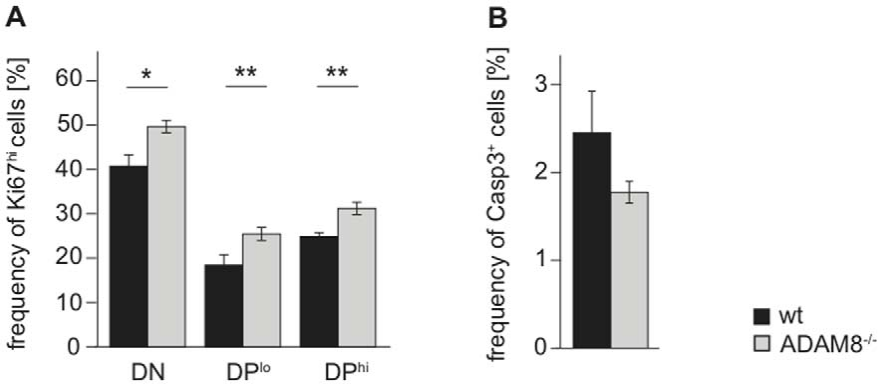
ADAM8^−/−^ thymocytes have increased proliferation and reduced apoptosis rates. (A) Frequencies of Ki-67^hi^ cells in DN, DP^lo^ and DP^hi^ subsets of wt and ADAM8^−/−^ thymi. (B) Frequency of Caspase-3^+^ thymocytes in wt and ADAM8^−/−^ thymi. Data are representative of two independent experiments with n≥4 age- and gender-matched mice per group in each experiment. Data are presented as mean values and error bars as SEM. *, p<0.05, **, p<0.01.

**Figure 6:**
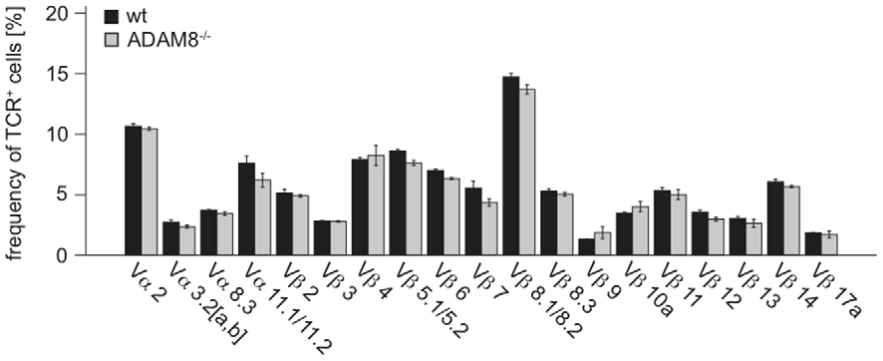
Similar usage of Vα and VβTCR chains in wt and ADAM8^−/−^ CD4 lymphocytes. Frequencies of CD3^+^ CD4^+^ lymph node cells expressing indicated subfamilies of Vα or Vβ TCR chains determined by flow cytometry. Data are representative of two independent experiments with n≥4 age- and gender-matched mice per group in each experiment. Data are presented as mean values and error bars as SEM.

An increased thymic cellularity can also result from a higher proliferation and/or reduced apoptosis. We therefore analyzed thymocytes from wt and ADAM8^−/−^ mice for the expression of Ki-67, a marker for proliferating cells [28], and activated Caspase-3, an apoptosis marker [29]. Frequencies of Ki-67^hi^ DN and DP thymocytes were significantly increased in ADAM8^−/−^ mice suggesting a higher proliferation rate (Figure 5A). Interestingly, there was also a substantial but not significant reduction in the frequency of thymocytes staining positively for activated Caspase-3 (Figure 4B). A reduced rate of cell death together with increased cell proliferation might thus contribute to thymic hyper-cellularity observed in ADAM8^−/−^ mice. The former could be a result of reduced negative selection whereas the latter of increased positive selection. However, analysis of the TCR repertoire in lymph nodes of wt and ADAM8^−/−^ mice did not show any skewing in the usage of the analyzed Vα and Vβ TCR subfamilies in ADAM8^−/−^ mice and therefore suggests that the thymocyte selection process in ADAM8^−/−^ mice is normal (Figure 6).

### Fibronectin-dependent migration is reduced in ADAM8^−/−^ thymocytes

**Figure 7:**
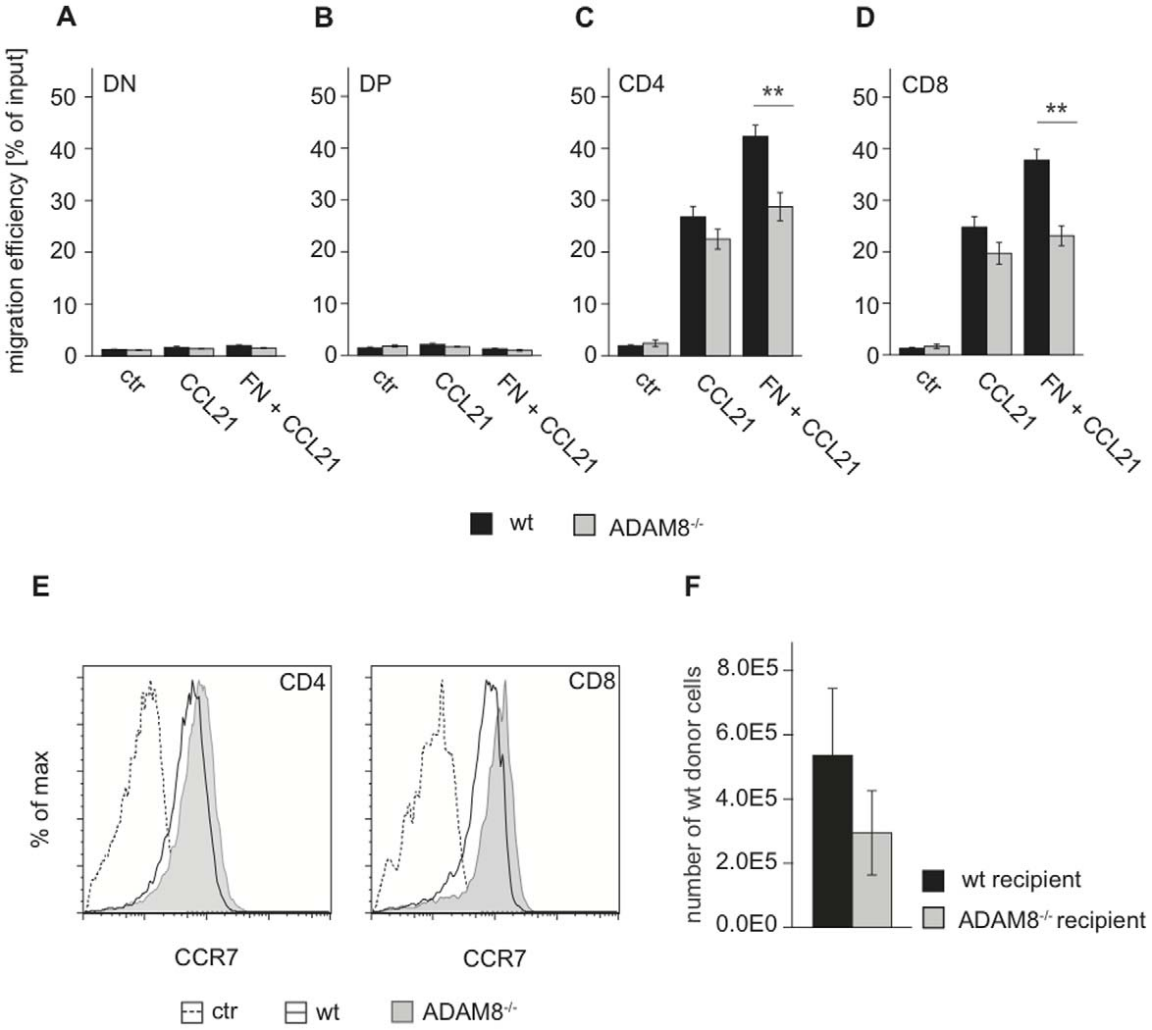
ADAM8^−/−^ thymocytes migrate less efficiently through matrix in response to CCL21. Migration efficiency [% of input] of wt and ADAM8^−/−^ (A) DN, (B) DP, (C) CD4 and (D) CD8 thymocytes in transmigration assays in response to CCL21 in the presence or absence of Fibronectin. (E) Flow cytometry plots showing CCR7 expression on wt and ADAM8^−/−^ CD4 and CD8 thymocytes detected with CCL19-Fc. (F) Numbers of donor-derived cells 10 days after intrathymic injection of wt bone marrow cells into wt or ADAM8^−/−^ mice. Data in (A–D) are representative of three independent experiments performed in triplicates. Data in (E) are representative of two independent experiments with n≥3 age- and gender-matched mice per group in each experiment. Data in (F) are representative of two independent experiments with n≥4 age- and gender-matched mice per group in each experiment. Data are presented as mean values and error bars as SEM. **, p<0.01.

ADAM8 deficiency has previously been associated with reduced migration of tumor cells [30], [31], [32] and several types of immune cells [33]. Since ADAM8 RNA levels were increased in mature thymocyte subsets, a defect in intrathymic migration to the medulla could also explain the accumulation of ADAM8^−/−^ SP thymocytes. Thus we tested the ability of ADAM8^−/−^ cells to migrate in a gradient of the chemokine CCL21 that is known to play a significant role in attracting thymocytes to the medulla [1], [4]. Chemotaxis assays carried out in transwell chambers revealed that ADAM8 deficiency did not affect the migration of DN and DP thymocytes. In contrast, ADAM8^−/−^ SP thymocytes showed a trend for lower migration efficiencies towards CCL21 when compared to wt controls (Figure 7A–D). In the presence of the ECM protein fibronectin however, we observed a statistically significant disadvantage in the migration efficiencies of ADAM8^−/−^ SP cells compared to wt controls. Importantly, differences in CCR7 expression between SP thymocytes of wt and ADAM8^−/−^ mice were not detected and thus could not account for differential responsiveness to its ligand CCL21 (Figure 7E).

Differences in migration and matrix interaction could cause ADAM8^−/−^ thymocytes to occupy certain thymic microenvironments for longer periods of time than thymocytes that express functional ADAM8 and as a result alter the availability of intrathymic niche space. To test this hypothesis, wt bone marrow cells were directly injected into the thymic lobes of wt and ADAM8^−/−^ recipient mice, and donor cells in the respective recipient thymi quantified 10 days later. Fewer wt donor cells engrafted in ADAM8^−/−^ recipient thymi compared to wt recipients suggesting that wt donor cells are less competitive in an ADAM8^−/−^ environment (Figure 7F).

## Discussion

ADAM8 expression in the thymus has previously been described [7], [8], [9], [10], [12], [13]. Here, we comparatively analyzed wt and ADAM8^−/−^ mice to identify in more detail the thymic cell subsets that express ADAM8 and to identify a potential function for ADAM8 in T cell development. We show that ADAM8 was expressed in thymic stromal cells and thymocytes and that ADAM8 RNA expression was most abundant in mTEC and in mature thymocytes. We found that absence of ADAM8 was associated with an accumulation of mature thymocytes and an increase in medulla size. These findings were unexpected as deletion of other ADAM family members resulted in a developmental block at the transition from DN to DP thymocytes [8], [9], [10] suggesting that ADAM8 functions in a non-redundant manner to these family members.

The thymus consists of several functionally different microenvironments that express unique sets of genes to support T cell development [1], [12]. It has been shown that the different microenvironments express different sets of adhesion and matrix molecules allowing only thymocyte subsets that express the proper ligands to enter these regions [4]. ADAM8 contains functional metalloproteinase and disintegrin/cysteine-rich domains with reported roles in ectodomain shedding [34] and integrin-disintegrin interaction [35]. ADAM8 expression was strongest in the medulla; hence this region was most affected in ADAM8^−/−^ mice, suggesting that ADAM8 binding partners and/or substrates modulate access and residence time of cells in the medullary microenvironment. The specific nature of the binding partners and/or substrates of ADAM8 however are still unknown.

ADAM8 has functionally been associated with tumor and immune cell migration [30], [31], [32], [33]. Studies on ADAM8 and other metalloproteinases revealed that metalloproteinase activity alone can promote cell attachment, cell outgrowth and cell migration [36], [37], [38], [39], [40] by either direct modification of ECM components such as fibronectin [36], or from proteolytic release of cell adhesion molecules that indirectly modify cell attachment and migration [37], [38], [39], [40]. Both mechanisms might be relevant for ADAM8-dependent intrathymic cell migration. The fact that ECM proteins such as fibronectin [41] and collagen I (Bartsch, personal communication) are proteolytically released by ADAM8 together with our finding that ADAM8^−/−^ thymocytes migrated less efficient through a fibronectin matrix suggests that direct modulation of matrix components by ADAM8 might be important for thymocyte migration. ADAM8-dependent intrathymic cell migration might furthermore be supported by proteolytic release of thymic cell adhesion molecules, e.g. in an integrin-dependent way as shown for ADAM10/L1 and [39] and suggested for ADAM8/Close Homologue of L1[40].

Maturation and expansion of mTEC have been shown to require direct interaction with mature thymocytes [42], [43]. The observed increase in medulla content in thymi of ADAM8^−/−^ mice is thus likely a secondary effect caused by the elevated number of mature T cells that are present in ADAM8^−/−^ thymi. Based on the observations that an ADAM8 deficient microenvironment reduced wt T cell progenitor engraftment compared to a wt microenvironment, we propose that local concentrations of thymocyte-intrinsic and extrinsic ADAM8 in the medulla are necessary for normal thymocyte migration and therefore normal development. Loss of ADAM8 expression impairs the migration efficiency of thymocytes in the microenvironment, leading to longer residence times and finally to accumulation of thymocytes and limited intrathymic niche space. These increased residence times might lead to longer thymocyte-TEC interaction providing the thymocytes with more survival and proliferation signals and thus explaining the observed increase in thymocyte proliferation and reduced apoptosis.

ADAM8^−/−^ mice do not have an obvious pathologic phenotype however we recently showed that the lack of ADAM8 renders mice more resistant to induction of experimental asthma and that this phenotype was in part T cell dependent [33]. We therefore propose that the role of ADAM8 is to support migration of immature and mature T cells.
